# West nile virus myocarditis causing a fatal arrhythmia: a case report

**DOI:** 10.1186/1757-1626-2-7147

**Published:** 2009-05-27

**Authors:** Anurag Kushawaha, Sunil Jadonath, Neville Mobarakai

**Affiliations:** Department of Medicine, Division of Infectious Diseases, Staten Island University Hospital475 Seaview Avenue, Staten Island, NY 10305USA

## Abstract

West Nile Virus is one of the most frequently reported etiologies of viral encephalitis in the USA. West Nile Virus infections among hospitalized patients manifests most commonly as neuro-invasive disease. West Nile Virus has also been reported to cause myocarditis. Arrhythmia is not an uncommon occurrence in viral myocarditis. As cases of West Nile Virus increase, it is important that the index of suspicion also increase for this uncommon complication. Physicians who are caring for West Nile Virus-infected patients need to be aware of the possibility of West Nile Virus -related myocarditis. The question arises whether a patient with an established diagnosis of West Nile Virus -meningoencephalitis should be under continuous cardiac monitoring, bearing in mind the rare, but fatal, complication of cardiac arrhythmia secondary to viral myocarditis. We present a case report of a 65-year-old man who initially presented with fever, blurry vision, and decreased oral intake who subsequently suffered a fatal arrhythmia; further laboratory tests and autopsy findings revealed the patient likely had developed encephalitis and myocarditis secondary to West Nile Virus infection.

## Case presentation

A 65-year-old man of Scottish-American descent presented to the emergency department (ED) in late summer 2008 complaining of fever, cough, and decreased oral intake for the past seven days. His symptoms were associated with blurry vision, nausea, and weakness. Past medical history included diabetes and hypertension. He was a former smoker (quit 4 years ago) and denied travel history or sick contacts.

In the ED, he was noted to be alert, conversant, and oriented. His temperature was 100.8 F, blood pressure was 133/76 mmHg, and heart rate was 104 bpm. Physical examination was unremarkable. The eyes were normal to inspection with normal visual acuity, extra-ocular movements were intact, with no evidence of scleral icterus or conjunctival erythema. Admission labs were notable for a sodium level of 125.2 mEq/L (reference range 135-146 mEq/L) and creatinine of 1.6 mg/dL (reference range 0.7-1.5 mg/dL). The WBC count was 7.6 × 10^3^/μL (reference range 4.8-10.8 × 10^3^/μL). Chest roentgenogram demonstrated no acute infiltrates. Electrocardiogram (EKG) showed normal sinus rhythm at a rate of 83. Blood cultures were drawn and the patient was started on intravenous ceftriaxone and azithromycin.

On day 1, the patient became slightly confused and disoriented at times. He still complained of blurry vision and was noted to have a fever of 103.4 F. Antibiotics were changed to ceftriaxone, vancomycin, and the antiviral acyclovir was also added.

On day 2, computed tomography of the head showed chronic inflammatory changes with no acute bleed, mass effect, or shift. Lumbar puncture was conducted and revealed colorless cerebrospinal fluid (CSF), 4 RBCs/mm^3^, 29 WBC/mm^3^ (68% lymphocytes, 31% neutrophils, 1% monocytes), glucose of 115 mg/dL (serum level, 219), and a protein of 195 mg/dL. Bacterial and fungal stains were negative. Herpes simplex virus (HSV) polymerase chain reaction (PCR) of the CSF was negative. Serum Lyme antibodies were negative. Blood cultures were negative. Vancomycin was stopped, while ceftriaxone and acyclovir were continued. A transthoracic echocardiogram revealed ejection fraction of 50-55% with abnormal left ventricular relaxation and a dilated left atrium. The serum sodium level was noted to be 128 mEq/L.

On day 3, the patient was noted to be lethargic and disoriented with a fever of 104.5 F. An electroencephalogram demonstrated abnormal moderate generalized background slowing consistent with diffuse encephalopathy. Antimicrobials were continued.

On day 4, during morning rounds, the patient was his usual self, but continued to be lethargic, yet arousable and responsive to questions, with no new complaints. Ten minutes later, he was found unresponsive and pulseless. The electrocardiogram revealed asystole. As the patient was not on telemetry monitoring nor was a routine electrocardiogram ordered for that morning, there was no other electrocardiogram to compare, besides the admission EKG. Cardio-pulmonary resuscitation was unsuccessful.

Subsequently, the CSF that had been sent to the Board of Health, was reported to be positive for West Nile Virus (WNV) genome by PCR. IgM antibodies for WNV were also reported positive in the CSF. Serum IgM antibodies for WNV were not carried out. An autopsy was conducted. The central nervous system examination revealed microglial nodules, sparse perivascular lymphocytic infiltrate, and focal leptomeningeal monovascular infiltrate consistent with viral meningoencephalitis. West Nile Virus genome was also detected in the brain tissue by PCR. The cardiovascular examination demonstrated focal left ventricular scarring with lymphocytes and histiocytes consistent with myocarditis. West Nile Virus genome was not detected in the myocardium by PCR. The autopsy revealed no evidence for pulmonary embolism or acute myocardial infarction.

## Discussion

The patient had WNV encephalitis with positive WNV genome by PCR in the CSF and brain tissue. The acute nature of the patient's decompensation pointed to a cardiac etiology. The autopsy showed no evidence of acute myocardial infarction or pulmonary embolism, but findings were consistent with viral myocarditis (although WNV genome by PCR was negative in the myocardium). Based on the autopsy findings and the lack of evidence for acute ischemia or infarct, viral myocarditis causing an acute arrhythmia was felt to be the most likely etiology for the demise of the patient.

Arrhythmia is not uncommon in viral myocarditis, including supraventricular tachycardia, atrial ectopic tachycardia, ventricular premature beats, ventricular tachycardia, and ventricular fibrillation [1]. Friedman et al., described 12 patients (ten patients < 18 years old), mostly without obvious myocarditis, who had biopsy findings consistent with myocarditis. Of these, eleven had ventricular tachycardia and one had multiform ventricular premature beats [2]. Wiles et al., made similar observations of 33 patients evaluated for ventricular ectopic rhythm using endomyocardial biopsy, three had focal lymphocytic myocarditis [3]. Take et al., described nine adults with complete heart block with viral myocarditis, which was permanent in two cases [4]. Currently, the “gold standard” for myocarditis is endomyocardial biopsy which unfortunately has low sensitivity and specificity [5].

There have been numerous case reports of myocarditis secondary to WNV in several mammalian species [6] and birds [7], indicating a predilection for myocardial involvement. These findings include multi-focal myocardial necrosis and lympho-histiocytic myocarditis [8]. Although the WNV genome PCR from the myocardium was negative, WNV myocardial involvement has only been described in few autopsy reports [9]. During a 1999 WNV epidemic in Russia, hydropericarditis with flabbiness of the cardiac muscle was described among 40 fatal cases. However, the number of patients with these cardiac findings and their ages were not specified [10]. Cardiac sequelae have been reported after infections with other flaviviruses [11].

The presence of neurologic involvement, positive WNV genome by PCR from the CSF and brain, and absence of other major viral infections, supports that WNV-associated myocarditis causing a fatal arrhythmia as the likely cause of the patient's demise. The implications of this case are significant. West Nile Virus is one of the most frequently reported etiologies of viral encephalitis in the USA. West Nile Virus infections among hospitalized patients manifests most commonly as neuro-invasive disease [12]. In recent outbreaks of WNV, overall mortality has been shown to be higher for the elderly, the presence of profound weakness, deep coma, failure to produce IgM antibody, impaired immunity, and coexisting illnesses, such as hypertension and diabetes mellitus [13] ( both of the latter conditions the patient had.) As cases of WNV increase, it is important that the index of suspicion also increase for this uncommon complication. Physicians who are caring for WNV-infected patients need to be aware of the possibility of WNV-related myocarditis. The question arises whether a patient with an established diagnosis of WNV-meningoencephalitis should be under continuous cardiac monitoring, bearing in mind the rare, but fatal, complication of cardiac arrhythmia secondary to viral myocarditis. Also, WNV should be considered with any case of acute myocarditis, particularly during the summer and fall when mosquitoes are most prevalent.

## List of abbreviations

bpm: beats per minute
CSF: Cerebrospinal fluid
ED: Emergency Department
EKG: Electrocardiogram
F: degrees Fahrenheit
HSV: Herpes simplex virus
mEq/L: milliequivalents per liter
mg/dL: milligrams per deciliter
mmHg: millimeters of mercury
PCR: Polymerase Chain Reaction
RBC/mm^3^: red blood cells per cubic millimeter
WBC/mm^3^: white blood cells per cubic millimeter
WNV: West Nile Virus
/μL: cells per microliter
